# Vesicovaginal fistula: An update

**DOI:** 10.4103/0970-1591.32073

**Published:** 2007

**Authors:** Santosh Kumar, Nitin S. Kekre, Ganesh Gopalakrishnan

**Affiliations:** Department of Urology, Christian Medical College, Vellore - 632 004, India

**Keywords:** Laparoscopy, robotic repair, vesicovaginal fistula

## Abstract

Vesicovaginal fistula (VVF) has been a social and a surgical problem for centuries and remains a challenge to surgeons. Though advances have been made in the understanding of the etiology, diagnostic procedures and management of these fistulae, controversies still exist over the ideal approach and time to repair. This review was undertaken to look into the recent literature with regard to the timing and surgical approach to VVF repair. The literature search was done using the Medline database with keywords: vesicovaginal fistula, laparoscopy and robotic repair filtered for the last 5 years.

Vesicovaginal fistula (VVF) has been a social and surgical problem for centuries. In 1935, while performing a dissection on an Egyptian mummy (a queen of the 11^th^ Dynasty, circa 2050 BCE), Derry noted a large VVF that he concluded was the consequence of obstructed labor.[1] Obstetric VVF related to prolonged labor remains a major medical problem in many underdeveloped countries with a low standard of obstetric care.[2] However, abdominal hysterectomy remains the most common cause of VVF in developed countries occurring in 1/1800 hysterectomies.[3] VVFs can appear 1-6 weeks after gynaecologic or obstetric surgery and recurrent fistulas can occur within 3 months of primary fistula repair.[2]

In 1852, James Marion Sims reported a successful repair of VVF in female slaves.[4] Inspite of there being several reports of successful fistula repair prior to his work, those descriptions were crude at best. Sims used silver wires as suture material, avoided the use of electrocautery and utilized postoperative bladder drainage with a urethral catheter. Initially, Trendelenberg[5] described transabdominal, transvesical repair which has become the standard treatment for difficult VVF.[6] Transvaginal repairs were introduced in an effort to decrease operative morbidity.[7] However, depending on the location and etiology of the fistulas, success rates for transvaginal repairs have been lower than those of transabdominal surgery.

VVFs that result from operative injury can be repaired with a success rate of 75-97%. A failure rate of 10% has been reported with recurrent fistulas.[8,9] VVF remains a challenge to the surgeon and a difficult, socially unacceptable nuisance to patients, more so if it is recurrent.[2] Controversy still exists over the timing, ideal surgical approach and need for adjuvant measures. This review was undertaken to look into the recent literature with regard to the timing and surgical approach to VVF repair. The literature search was done using the Medline database with keywords: vesicovaginal fistula, laparoscopy and robotic repair filtered for the last 5 years.

## EARLY *VS* DELAYED REPAIR

The appearance of a fistula between the bladder and the vagina is one of the most devastating postoperative complications of some gynecological procedures. The emotional distress of the patient and surgeons is high because of the little hope that conservative therapy offers and the need for a second operation to correct the problem. The most commonly followed practice is to provide a trial of conservative therapy with proper and undisturbed bladder drainage, antibiotics when indicated and for the small fistulas, an attempt to fulgurate the area. Some success has been reported (7-12.5%) in select cases.[2,10] Transvesical and / or transvaginal use of glue injected through a cystoscope has been described for small fistulas. Fibrin glue is attractive as a tissue sealant because it prevents fibrosis and promotes healing through its effects on fibroblasts and collagen synthesis. It is biodegradable, being absorbed within weeks of its application. The first series[11] describing the use of fibrin glue for VVFs demonstrated a 66% success rate in 6 patients as compared to 88% with traditional repair. Since then, there have been many series reporting success but all with small numbers of patients.[12,13] Fibrin glue has also been used in failed repairs and patients who had radiation therapy and chemotherapy.[14] Sometimes, VVF repair has failed after 4 years, necessitating a urinary diversion. When conservative treatment has failed and leakage of urine continues per vaginum despite good bladder drainage, surgical correction of the fistula becomes necessary.

The timing of surgery remains a controversial issue. Presence of infection of the vaginal cuff or pelvic infection requires a prolonged antibiotic therapy before any attempt at repair. The classical opinion on timing the surgery is to wait for 3-6 months to allow the surgical inflammatory reaction to subside. Shortening the waiting period to undergo VVF repair surgery is socially and psychologically important in these already distressed patients. However these reasons should be carefully weighed against the risk of a compromised success.

There is no consensus as to the definitions of late and early repair with the definition of early repair ranging from 1-3 months and that of late ranging from 2-4 months after diagnosis of VVFs in different series. In series in which all repairs were done early,[15,16] success rates ranged from 86 to 100% although the patient numbers were small (7 and 11 respectively). Series in which all repairs were considered late, success rates ranged from 88-94%.[17,18] Once acute inflammation has subsided, there is no benefit to be derived from delaying surgery. Delay has a devastating impact on the quality of life and ability to work for these unfortunate patients. Delaying surgery until infection has subsided has advantages mainly in the case of obstetric fistulas. The neck of the bladder and the proximal urethra are the most common locations of VVFs and are best repaired vaginally. Local sloughing and healing in this situation should be allowed to “declare” the fistula site and health of the urethra. Early repairs may result in morbidity but their risks are usually outweighed by the social higher stigma of living with a fistula and a perineum bathed in urine. To conclude, mandating all patients to delayed repairs does not appear to result in statistically superior results and may have significant social and psychological ramifications for the woman and her family.[19]

## ABDOMINAL *VS* VAGINAL APPROACH

The first operation has the highest success rate to repair a VVF. Surgeons differ in their approach to repair these fistulas, taking into consideration the cause, location, size and time of onset. The selected route of repair depends mostly on the training and experience of the surgeon. The best approach is probably the one in which the surgeon is most experienced. Most surgeons favor either a vaginal or an abdominal approach and exclusively report on those techniques. Those who report on both techniques generally do so by espousing individual preference and reviewing their experience at their respective institutions.[19]

When VVFs result from difficult hysterectomy, initial repair is usually attempted via a vaginal approach, most often by gynecologic surgeons. This approach is satisfactory in most cases.

The vaginal approach avoids laparotomy and splitting of the bladder and recovery is shorter with less morbidity, blood loss and postoperative bladder irritability. The procedure can be done in an outpatient setting; postoperative pain is minimal and results as successful as those of the abdominal approach are achieved.[20] Exclusion criteria for vaginal repair are circumferential induration at the fistula site exceeding 2 cm, fistula location or vaginal architecture precluding adequate vaginal exposure, fistulas involving the ureters or the patient's preference for an abdominal approach after preoperative counseling.

The two principal techniques for the transvaginal approach are the Latzko and the vaginal flap techniques.[7,21] The Latzko procedure (partial colpocleisis) has been popular for the repair of small fistulas while the vaginal flap technique is used for larger ones. A Martius (labial fat pad) flap is used to provide a second layer of tissue over the fistula repair. If a single labial fat pad flap does not provide adequate coverage, a second flap may be obtained from the other side. Neither procedure involves excision of the fistulous tract. The disadvantages of the Latzko techniques are that it potentially shortens the vagina and cannot be performed when the uterus is present. A potential shortcoming of the vaginal flap technique is excessive tissue mobilization, which can result in avascular necrosis at the suture lines or incorporation of the ureters into the closure.[22] Success rates ranging from 82-94% have been reported for the transvaginal approach.[2,16,22]

Principles of surgical repair of VVF include optimal tissue condition (adequate vascular supply and freedom from infection, inflammation, necrosis and malignancy), option of complete excision of fistulous tract, a tension-free, water-tight, multilayered closure with avoidance of overlapping suture lines, interposition of healthy vascularised tissue between the bladder and vaginal suture lines and continuous postoperative bladder drainage. Transabdominal repair described by O'Conor adheres to these guiding principles.

The omentum which is usually used for interposition, has an abundant vascular supply and lymphatic drainage. It provides the suture lines with a vascular graft, replacement tissue and a mechanism for absorption of debris increasing the chance of success of the repair.[23] After healing, the omentum retains its suppleness and maintains a plane of separation, should reoperation be necessary.

The abdominal approach is indicated in 1. Inadequate exposure related to a high or retracted fistula in a narrow vagina. 2. Close proximity of the fistulous tract to the ureter. 3. Associated pelvic pathology requiring simultaneous abdominal surgery and 4. Multiple and recurrent fistulas 5. Supratrigonal location 6. Surgeon's inexperience with vaginal surgery.

In complicated cases, a combined transabdominal and transvaginal approach has been reported.[24] The abdominal approach has enjoyed reproducible and durable success from 94-100%.[24,25] The use of limited anterior cystotomy has improved the historically more morbid O'Conor procedure in which the bladder is bivalved to the level of the fistula.

## LAPAROSCOPIC VVF REPAIR

Laparoscopic VVF repair attempts to achieve success rates similar to those of transabdominal repair and avoids the morbidity of open surgery. Since first reported by Nerzhat in 1994,[26] there are many case reports of laparascopic VVF repair and a few case series which are summarized in Table 1.

**Table 1:** Case reports of laparascopic vesicovaginal fistula repair

References	No. Pts	Mean operative time (mins)	Mean blood loss (CC)	Mean hospital stay (days)	Mean foley-catheter duration (days)
Nezhat *et al*[26]	1	85	100	1	10
Phipps[27]	2	160	Data NA	1	10
Von Theobald *et al*[8]	1	70	100	8	7
Miklos *et al*[28]	1	Data NA	Data NA	1	21
Nabi and Hemal[29]	1	190	Data NA	4	21
Ou *et al*[30]	2	Data NA	Data NA	2-12	14-20
Chibber *et al*[31]	8	220	Data NA	3	14-21
Wong *et al*[32]	2	381	< 100	2	21
Sotelo *et al*[33]	15	170	Data NA	3	10.4
Modi *et al*[34]	1	170	50 ml	2	14

The procedure is done under general anesthesia. The patient is first placed in low lithotomy and cystoscopy is performed to see the site, size, number of fistulas. Both ureters are catheterized cystoscopically to facilitate ureteral identification and protection during excision and closure of the fistula. A ureteric catheter (if the fistula is small) or a Foley's catheter is passed through the VVF and retrieved through the vagina to facilitate identification and dissection. A sponge stick inserted into the vagina and if required, a cystoscope in the bladder (or a fluid-filled bladder with the catheter clamped) aids in laparoscopic dissection.

For laparoscopy, the patient is placed in the Trendelenberg position. A 5 or 4 port transperitoneal approach is used (a 12 mm infraumbilical trocar, two additional 10 mm trocars in the right and left lower quadrants and a 5 mm suprapubic trocar). The peritoneum over the bladder is incised transversely and the bladder opened vertically down up to the fistula. Stay sutures may be placed at bladder edges for exposure. The bladder is separated from the vagina, completely excising the fistula margin and adjacent fibrotic tissue. The bladder and vagina are closed separately with a single layer of full thickness-interrupted 2-0 Vicryl sutures. An omental flap or pericolic fat is interposed between the bladder and vaginal suture lines. A pelvic drain is left though one of the ports. A gravity cystogram is performed 2-3 weeks post operatively and if it is normal, the ureteral catheter is removed.

The advantages of a minimally invasive procedure include magnification during the procedure, hemostasis, decreased abdominal pain and a shorter hospital stay with quicker recovery and early return to work. In addition to providing excellent exposure, the laparoscopic approach allows easy mobilization of the omentum for interposition. Laparoscopic VVF repair adheres to the principles of transabdominal VVF repair while decreasing morbidity and improving cosmesis. Laparoscopic VVF repair is a feasible and efficacious approach with a successful outcome in a majority of the patients. However laparoscopic forehand intracorporeal suturing can be a challenging task.[33]

## ROBOTIC VVF REPAIR

Laparoscopic surgery has been developed by urologists over the past two decades. The number and complexity of surgical procedures performed is ever growing. Still, however, technical limitations exist, causing steep learning curves for many laparoscopic procedures, especially those in the pelvis. Laparoscopy poses immense technical difficulty in VVF repair particularly in dissecting out the fistulous tract and in applying sutures to repair the bladder and vagina separately with omentum interposition.[29] Due to these technical difficulties, laparascopy has not gained widespread popularity and has been limited to some centers.[35]

Using robotic surgical systems to perform minimally invasive procedures offers many potential advantages. The da Vinci robotic surgical system consists of a free-standing robotic tower and a console. The robotic tower has a camera arm and two or three instrument arms. The console provides a 6-12X magnified three-dimensional (3D) image of the surgical field and an ergonomically designed interface. Sitting comfortably at the console, the surgeon can manipulate the hand controls and view the operation live through the Insight vision system. This helps to translate small hand and wrist movements via EndoWrist-articulated instruments with seven degrees of freedom and bidirectional articulation plus grip, into precise movements inside the body. The assistance of the robot reduces operating fatigue and filters out unpredictable movements and tremors inherent in human hands.[36]

The first report of robot-assisted laparoscopic repair of a VVF was from the University of California, Irvine in a 44 year-old woman with a small VVF after vaginal hysterectomy.[37] Cystoscopy and single J catheters were placed initially. Laparoscopy was done in extreme Trendelenberg position. Five ports were placed and dissection was done in the standard fashion and the fistula tract excised. The da Vinci robotic system was then docked to close the vagina in a single layer and bladder in two layers. Fibrin glue was injected between the bladder and vagina to separate the suture lines. The total operating time was 280 minutes and estimated blood loss was 50 ml. The patient was discharged on the second postoperative day and voided normally after the catheter was removed 2 weeks postoperatively.

Sundaram **et al** published the first series of five patients who underwent robotic repair of VVF.[38] The etiology was posthysterectomy in four patients and postmyomectomy in one. After the initial cystoscopic examination, five ports were placed as described for pelvic robotic surgery in women.[39] One patient had a cystoscopic ureteric catheterization and another had a perioperative robot-assisted bilateral ureteric catheterization. VVF repair was done in the standard fashion and both vagina and bladder closed in two layers, the vagina was closed horizontally and the bladder vertically after omental interposition. The mean size of VVF was 3.1 cm and mean time of surgery was 233 minutes with an estimated blood loss < 70 ml. Patients were ambulated the same day and mean hospital stay was 5 days. Catheter was removed on the 10^th^ postoperative day after voiding cystourethrography. All patients had a successful outcome with no recurrence seen at the 6 month follow-up.

The 3-D magnified superior view of the daVinci allows successful identification of the correct tissue planes for appropriate dissection. The robot helps tremendously in suturing, which is an arduous task during routine laparoscopy. Robotic assistance offers technical advantages during complex laparoscopic repair of VVF. However, the cost of procuring a robotic system is high and the recurrent cost of consumables is the main hindrance to its routine use in most centers.

## CONCLUSIONS

VVF continues to be a social and surgical problem. Obstetric VVF remains a problem in underdeveloped countries whereas abdominal hysterectomy is the most common cause of VVFs in developed countries. The timing and route of surgical repair are best tailored to the individual treating surgeon. Mandating all patients to delayed timing of repair or any one technique does not appear to result in statistically superior results and may have significant social and psychological ramifications for the woman and her family.

The laparoscopic approach to VVF repair is an excellent alternative to the traditional abdominal approach but it requires laparoscopic experience, particularly pelvic surgery with intracorporeal suturing. The daVinci Robot system provides a 3-D magnified superior view and improved coordination which helps make the dissection and suturing simpler. As these new technologies continue to develop, the shortened learning curve they provide will likely allow an increased number of urologists to provide their patients with technically proficient and less invasive operations.
